# The military occupational stress response scale: Development, reliability, and validity

**DOI:** 10.3389/fpsyg.2023.1032876

**Published:** 2023-02-16

**Authors:** Qianwen Wu, Zhibing Yang, Rui Qiu, Sizhe Cheng, Xia Zhu, Zheyi Han, Wei Xiao

**Affiliations:** ^1^Department of Military Medical Psychology, Air Force Medical University, Xi'an, China; ^2^Department of Gastroenterology, Air Force Medical Center PLA, Beijing, China

**Keywords:** military personnel, occupational stress, stress response, reliability, validity

## Abstract

Soldiers in the military are exposed to numerous stressors, including some that are of an extreme nature. The main objective of this military psychology research study was to evaluate soldiers' occupational stress. Even though several tools have been developed to measure stress in this population, to date, none have focused on occupational stress. Hence, we developed the Military Occupational Stress Response Scale (MOSRS) to provide a tool to objectively measure soldiers' occupational stress responses. An initial pool of 27 items was assembled from the literature, existing instruments, and interviews with soldiers. Of those 27, 17 were included in the MOSRS. The scale was subsequently completed by soldiers from one military region, and exploratory factor analysis (EFA) and confirmatory factor analysis were conducted using Mplus8.3 and IBM SPSS 28.0 software, respectively. A total of 847 officers and soldiers were selected for scale testing, and 670 subjects were retained after data cleaning and screening according to the set criteria. After performing the Kaiser–Meyer–Olkin (KMO) and Bartlett's test, principal components analysis (PCA) was appropriate. The PCA yielded a three-factor model (physiological, psychological, and behavioral responses) with the items and factors strongly correlated. The confirmatory factor analysis revealed loads ranging from between 0.499 and 0.878 for each item. The Cronbach's α coefficient of the MOSRS was between 0.710 and 0.900, and the Omega reliability was between 0.714 and 0.898, which were all higher than the critical standard value of 0.7, indicating that the scale has good reliability. Analysis of the discrimination validity of each dimension revealed that the scale has good discrimination validity. The MOSRS demonstrated sound psychometric characteristics with acceptable reliability and validity, suggesting that it could be used to assess occupational stress in military personnel.

## 1. Introduction

The military profession exposes personnel to a large number of stressors, including some that are very extreme and more intense than those experienced in other human activities. While the research category of extreme combat stress reactions causing casualties has not attracted attention from psychologists, it has attracted great attention from psychiatrists. Military psychiatrists primarily treat and permit the wounded members to return to work. A review of the literature found that military stress research focuses heavily on the fields of combat stress and post-traumatic stress disorder (PTSD) and less on daily stress from military occupations.

Regarding the relationship between occupational stress and physical and mental health, numerous studies have been focused in the same direction, examining the close relationship between occupational stress and physical health. Kawakami and Tsutsumi (2010) summarized the relationship between occupational stress and physical and mental health in recent years and found that different stress levels resulted in different stages of physical and mental health among various professions. The military is a high-risk, stressful career, and it is necessary to develop a pressure scale suitable for measuring the occupational effectiveness of soldiers.

The question of how to objectively and accurately evaluate these factors should form the basis of further study on the relationship between stress and work environment, performance, and physical and mental health. There is also a need to compare different occupational stress levels and take effective intervention measures to optimize the work stress level. A universally applicable, objective, and accurate assessment tool needs to be established to assess the stress experienced by military personnel.

Based on the study of occupational stress in military personnel in their home country and abroad, foreign military personnel most commonly use occupational stress questionnaires intended for the general population, such as the Occupational Stress Inventory—Revised (OSI-R) (Osipow and Spokane, 1998) and the Perceived Stress Scale (PSS) (Cohen et al., 1983). Luo et al. (2012a) have compiled The Psychological Stressors of Soldiers in the Southern Theater Command scale, however, it ignores the influence of military stressors.

This study takes military career stress as the entry point and aimed to provide scientific tools for measuring and evaluating military career stress and lay the foundation for the development of research on military career stress. Through investigation of the risk factors and protective factors affecting military occupational health, the military daily stress event database was established, existing occupational stress scales and theories were then borrowed, and tools for measuring daily occupational pressures were compiled. Its reliability and validity have also been preliminarily verified.

## 2. Methods

### 2.1. The military occupational stress hypothesis

**Figure 1:**
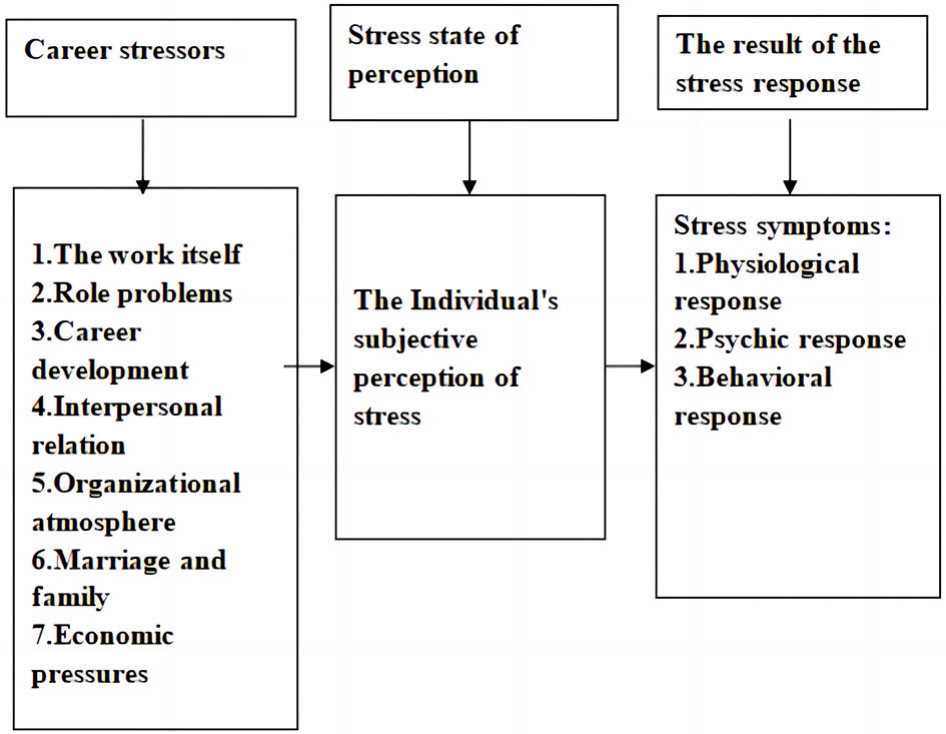
Structural chart of military occupational stress.

In the previous general occupational stress model, occupational stressors include work itself, the role in the organization, career development, work of interpersonal relationships, and the organizational structure and atmosphere (Greenberg, 2006), based on the professional characteristics of soldiers. Based on a literature review, which actively gathered information on the advanced experiences and achievements of domestic and foreign military occupational stress research and measurement, this study puts forward a military occupational stress structure diagram, shown in Figure 1.

### 2.2. Scale question bank preparation

On the basis of the literature review, military stress events were investigated and in-depth interviews were conducted to establish a database of military occupational stress events. In the previous stage of the prediction scale preparation, 22 officers and soldiers (15 men and 7 women and 9 officers and 13 soldiers) were selected using the convenience sampling method for interviews. The entries and sentences of related stressors were extracted from the stress event database and compiled based on the occupational stress structure of the military inside and outside of the military.

### 2.3. Objectives

A total of 847 soldiers were selected to test the scale, and 670 subjects were retained after cleaning and screening according to the standards set in the previous stage. Among them, 335 were randomly selected for exploratory factor analysis (EFA) (sample 1 data), and 335 were randomly selected for confirmatory factor analysis (sample 2 data). According to the data cleaning and screening criteria, subjects with response times of <500 s and those who answered linearly and regularly were excluded.

#### 2.3.1. Sample 1

A total of 335 subjects were included in sample 1. This data included information on the age of personnel, ranging from between 17 and 42 years (22.97 ± 4.263 years), and the length of military service, which ranged from 1 to 24 years (1.10 ± 0.294 years). The sample included 303 men (90.4%) and 32 women (9.6%) who were divided into the following groups: conscripts, noncommissioned officers, and officers, of which two did not fill in the category of rank. There were 161 conscripts (48.1%), 126 non-commissioned officers (37.6%), and 46 officers (13.7%). Their education level were divided into the following five categories: junior high school, senior high school, junior college, bachelor's degree, and master's degree or above, among which five people did not fill in the education level category. There were 43 junior high school students (12.8%), 158 senior high school students (47.2%), 73 junior college students (21.8%), 52 undergraduate students (15.5%), and 4 students with master's degrees or above (1.2%).

#### 2.3.2. Sample 2

Sample 2 included 335 subjects, including data on the age range of the personnel, which ranged between 17 and 34 years (22.69 ± 3.412 years) and the length of military service, which was between 1 and 15 years (3.87 ± 3.354 years). There were 289 men (86.3%) and 46 women (13.7%) whose grades were categorized into conscripts, non-commissioned officers, and officers, one of which did not fill in the grade category. There were 178 conscripts (53.1%), 118 non-commissioned officers (35.2%), and 38 officers (11.3%). Their education levels were divided into the following five categories: junior high school, senior high school, junior college, bachelor's degree, and master's degree or above. There were 42 junior high school students (12.5%), 154 senior high school students (46.0%), 95 junior college students (28.4%), 40 undergraduate students (11.9%), and 4 students with master's degrees or above (1.2%).

## 3. Procedure

The effects of the occupational stress response were divided into physiological response, psychological response, and behavioral response. The items of the occupational stress response scale for military personnel were chosen as follows: some occupational stress response items were taken from existing theoretical research literature; items from existing stress response scales that were suitable for the actual situation of the military arms were extracted or rewritten appropriately; and items were developed according to the results of the interview outline.

Microsoft Excel was used for data entry. After data entry, Statistical Package for the Social Sciences (SPSS) version 28.0 (IBM) was used to manage, describe, statistically analyze, and project the data of sample 1, and EFA was conducted. The confirmatory factor analysis was performed on the data of sample 2 using Mplus8.3.

## 4. Results

The scale was set to four points from 0 to 3. The higher the score, the greater the pressure value. There was no reversal clause. Descriptive statistical analysis was used to analyze the items in terms of minimum and maximum, average, standard deviation, kurtosis, and skewness.

As shown in Table 1, except for R11 and R12, the minimum and maximum values of the items were 0 and 3, respectively, which indicates that the questionnaire has a good range of items. On the concentration trend, the average value of the items was between 0.39 and 1.57; on the discrete trend, the standard deviation was between 0.609 and 0.966. According to correlation analysis, the correlation between the items and the total score was between 0.399 and 0.716, which met the requirements. According to the independent sample *t*-test, the decision value of each item was between 7.541 and 19.396, which is higher than 3 and meets the requirements.

**Table 1:** Analysis of the characteristics of each item in the scale.

**Item**	**Minimum**	**Maximum**	**Average**	**SD**	**Frequency**				***t***	***r***
					**0**	**1**	**2**	**3**		
R1	0	3	1.46	0.79	42	117	156	20	13.277	0.596^***^
R2	0	3	0.99	0.79	99	147	83	6	17.899	0.716^***^
R3	0	3	0.94	0.81	112	141	73	9	13.931	0.644^***^
R4	0	3	0.85	0.81	132	129	67	7	18.687	0.736^***^
R5	0	3	0.85	0.81	129	135	62	9	15.735	0.669^***^
R6	0	3	1.04	0.85	104	125	95	11	17.110	0.708^***^
R7	0	3	1.47	0.84	51	101	157	26	10.728	0.551^***^
R8	0	3	0.57	0.74	191	100	41	3	14.283	0.675^***^
R9	0	3	0.51	0.68	199	102	33	1	15.723	0.690^***^
R10	0	3	0.39	0.61	224	93	16	2	14.301	0.692^***^
R11	0	2	0.51	0.71	205	88	42	0	17.586	0.710^***^
R12	0	2	0.48	0.66	205	99	31	0	19.396	0.711^***^
R13	0	3	0.51	0.72	207	86	41	1	17.596	0.698^***^
R14	0	3	0.90	0.97	156	74	87	18	9.562	0.489^***^
R15	0	3	0.79	0.79	142	124	65	4	12.420	0.614^***^
R16	0	3	1.04	0.81	97	137	93	8	10.947	0.560^***^
R17	0	3	1.57	0.88	54	72	174	35	7.541	0.399^***^

*** *p* < 0.001.

As shown in Table 2, after performing the KMO and Bartlett's test at a *p*-value of < 0.01, the difference was extremely significant, with a KMO value of 0.918, close to 1, allowing for PCA.

**Table 2:** KMO and Bartlett's test.

Kaiser–Meyer–Olkin measure of sampling adequacy		**0.918**
Bartlett's test of sphericity	Approximate Chi-square (χ^2^)	2,817.855
	Df	136.000
	Sig.	0.000

Table 3 is designed to measure the correlation of the terms in the 3 components. Component 1 is the physiological response, component 2 is the psychological response, and component 3 is the behavioral response.

**Table 3:** Rotated component matrix for principal components analysis (PCA) with varimax rotation of three-factor solution with 17 items (*N* = 335).

**Item**	**Component 1**	**Component 2**	**Component 3**	**Communalities**
R1	0.152	**0.592**	0.253	0.437
R2	0.359	**0.673**	0.131	0.599
R3	0.259	**0.722**	0.022	0.589
R4	0.288	**0.719**	0.214	0.646
R5	0.265	**0.700**	0.112	0.573
R6	0.161	**0.771**	0.244	0.679
R7	0.111	**0.583**	0.216	0.399
R8	**0.780**	0.222	0.117	0.672
R9	**0.720**	0.340	0.062	0.638
R10	**0.812**	0.180	0.187	0.727
R11	**0.836**	0.222	0.131	0.766
R12	**0.823**	0.238	0.126	0.749
R13	**0.740**	0.263	0.173	0.647
R14	0.132	0.203	**0.583**	0.399
R15	0.385	0.172	**0.613**	0.554
R16	0.171	0.205	**0.736**	0.613
R17	−0.015	0.123	**0.706**	0.514
Eigenvalue	7.167	1.762	1.272	
Sums of squared loadings	42.16%	52.52%	60.01%	

The meaning of the bold values mean terms related to the factors.

Confirmatory factor analysis showed that χ^2^ = 2.247, root mean square error of approximation (RMSEA) = 0.061, 95% confidence interval (CI) (0.051–0.071), comparative fit index (CFI) = 0.942, Tucker–Lewis index (TLI) = 0.932, standardized root mean square residual (SRMR) = 0.048, and model fit indicators have a good fit, indicating that the Military Occupational Stress Response Scale (MOSRS) has good structural validity.

As shown in Table 4, the average variance extracted (AVE) values of psychological and behavioral responses are not higher than the critical standard of 0.50. However, the composite reliability (CR) values were all higher than 0.70, which indicates good convergence validity.

**Table 4:** Confirmatory factor analysis of the military occupational stress response scale (MOSRS).

**Item**	**Loading**	**SE**	***Z***	***p***	**Standardized factor loading**	**CR**	**AVE**
**Psychological**						0.858	0.465
R1	1	–	–	–	0.678		
R2	1.163	0.095	12.185	<0.001	0.758		
R3	1.096	0.097	11.263	<0.001	0.685		
R4	1.102	0.099	11.116	<0.001	0.697		
R5	1.066	0.097	11.032	<0.001	0.687		
R6	1.137	0.102	11.202	<0.001	0.704		
R7	0.846	0.093	9.069	<0.001	0.546		
**Physiological**						0.890	0.578
R8	1	–	–	–	0.635		
R9	1.100	0.100	10.983	<0.001	0.692		
R10	0.983	0.091	10.808	<0.001	0.685		
R11	1.354	0.109	12.379	<0.001	0.834		
R12	1.362	0.107	12.774	<0.001	0.878		
R13	1.370	0.113	12.118	<0.001	0.806		
**Behavioral**							
R14	1	–	–	–	0.563	0.723	0.401
R15	0.944	0.110	8.545	<0.001	0.686		
R16	1.117	0.137	8.178	<0.001	0.754		
R17	0.667	0.102	6.513	<0.001	0.499		

SE, standard error; CR, composite reliability; AVE, average variance extracted.

As shown in Table 5, the correlation coefficient between the dimensions was between 0.389 and 0.607, and the square root of AVE was higher than the correlation coefficient between dimensions, which indicates that it has good discriminant validity.

**Table 5:** Analysis of correlation and discriminant validity of each dimension.

**Factor**	**Mean**	**SD**	**Psychological**	**Physiological**	**Behavioral**
Psychological	1.200	0.620	**0.682**		
Physiological	0.570	0.600	0.607^***^	**0.760**	
Behavioral	1.210	0.650	0.474^***^	0.389^***^	**0.633**

*** *p* < 0.001. SD, standard deviation.

The meaning of the bold values mean the square root of AVE.

As shown in Table 6, The Cronbach's α coefficient of the dimension scale was between 0.710 and 0.900, and the Omega reliability was between 0.714 and 0.898, which are all higher than the critical standard of 0.7, indicating that the scale has good reliability.

**Table 6:** Reliability analysis of each dimension.

**Factor**	***N***	**Cronbach α**	**Omega**	**Split-half**
Psychological	7	0.856	0.856	0.795
Physiological	6	0.889	0.890	0.840
Behavioral	4	0.710	0.714	0.653
Overall	17	0.900	0.898	0.810

## 5. Discussion

In this study, the factor structure of a new scale—the MOSRS—was explored through exploratory and confirmatory factor analyses. The results showed that the scale included the following three factors: a physiological response, a psychological response, and a behavioral response. Physiological responses mainly included physical symptoms and physical discomfort caused by occupational stress and were biased toward physiological and pathological symptoms. Psychological responses primarily included cognitive symptoms and negative emotions resulting from work stress, while behavioral responses mainly focused on behaviors and negative styles of coping with work stress.

PCA supported the three-factor model of the MOSRS and showed that the factor structure was clear and had high structural validity. The three-factor model was created based, in part, on the research of Schmitt (1987), who reported that the theoretical hypothesis should be considered in addition to any results obtained from the data when choosing the model type. In our study, all results including Cronbach's α and Omega of the total scale, correlation, and discrimination validity were acceptable. Therefore, they may be used as effective indicators to measure occupational stress responses among military personnel.

In the past, stress scales for soldiers have focused on external stressors including events and effects in the environment rather than on subjective and internal feelings (Fevre et al., 2017; Wang et al., 2018), such as one's work environment, marriage, and relationships with family and friends. In comparison, the scale we developed focuses solely on the stress response. However, this scale can be used separately and together with other stressor scales for a comprehensive assessment of military occupational stress. Richard and Susan (1984) believed that one of the key parts of an individual's response to a stressful event is “cognitive evaluation” (Vallerand and Reid, 1984), which is partly dependent on an individual's assessment of his/her ability to respond to the event themselves (Lazarus, 2014). The “black box” that lies between stressors and stressful feelings is known as cognitive evaluation, which ultimately influences an individual's response to stress, together with stressful feelings. It ignores individual differences in the stress response and individual and situational factors and only measures military occupational stressors. The MOSRS proposed in our study differs from previous military occupational stress scales (Yao and Zhang, 2008; Luo et al., 2012b). In this study, the MOSRS scale was used to evaluate soldiers' responses to stress, that is, the subjective reaction that lies between being stimulated by a stressor and resulting in stressful feelings. The identification of these stress responses is the first step toward developing effective interventions to reduce the negative effects of stress on soldiers' military work performance.

Although the development of the scale has achieved good reliability and validity, which reflects occupational stressors and occupational stress responses, there are still some limitations of this study that we acknowledge and additional areas of research to be explored are as follows:

First, the subject group selected for this study was relatively homogenous, and only officers and soldiers of one military department participated. Hence, it is not known how generalizable our findings are to other populations. In the future, additional research using the MOSRS should be undertaken with other military arms and divisions to further evaluate the scale's value and effectiveness.

Second, after objectively and effectively evaluating the professional stresses faced by soldiers, measures should be taken to guide officers and soldiers in conducting effective stress management. Future studies, which are outside the scope of the current study, may develop military occupational stress assessment manuals and establish officers' and soldiers' occupational stress files, according to different occupational pressure levels. In addition, effective intervention measures may be implemented to maximize the potential of the military workforce in their careers, improve the officers' and soldiers' management of stress and their stress responses, and optimize their work performance and physical and mental health.

## Data availability statement

The raw data supporting the conclusions of this article will be made available by the authors, without undue reservation.

## Ethics statement

Ethical review and approval was not required for the study on human participants in accordance with the local legislation and institutional requirements. The patients/participants provided their written informed consent to participate in this study.

## Author contributions

QW and ZY: conceptualization, methodology, software, investigation, formal analysis, and writing—original draft. RQ: data curation, visualization, and investigation. SC: resources and supervision. XZ: conceptualization, funding acquisition, resources, supervision, and writing—review and editing. ZH and WX: supervision and writing—review and editing. All authors contributed to the article and approved the submitted version.
